# Evaluation of cough peak expiratory flow as a predictor of successful mechanical ventilation discontinuation: a narrative review of the literature

**DOI:** 10.1186/s40560-017-0229-9

**Published:** 2017-06-02

**Authors:** Chuan Jiang, Antonio Esquinas, Bushra Mina

**Affiliations:** 1 Department of Medicine, Northwell Health, Lenox Hill Hospital, New York, NY USA; 2 Intensive Care and Non-Invasive Ventilatory Unit, Hospital Morales Meseguer, Murcia, Spain; 3 Department of Medicine, Pulmonary and Critical Care Medicine, Northwell Health, Lenox Hill Hospital, New York, NY USA

**Keywords:** Mechanical ventilation, Respiratory failure, Weaning, Extubation, Spontaneous breathing trial, Cough strength

## Abstract

A crucial step in the transition from mechanical ventilation to extubation is the successful performance of a spontaneous breathing trial (SBT). The American College of Chest Physicians (ACCP) Guidelines recommend removal of the endotracheal tube upon successful completion of a SBT. However, this does not guarantee successful extubation as there remains a risk of re-intubation. Guidelines have outlined ventilator liberation protocols, selected use of non-invasive ventilation on extubation, early mobilization, and dynamic ventilator metrics to prevent and better predict extubation failure. However, a significant percentage of patients still fail mechanical ventilation discontinuation. A common reason for re-intubation is having a weak cough strength, which reflects the inability to protect the airway.

Evaluation of cough strength via objective measures using peak expiratory flow rate is a non-invasive and easily reproducible assessment which can predict extubation failure. We conducted a narrative review of the literature regarding use of cough strength as a predictive index for extubation failure risk. Results of our review show that cough strength, quantified objectively with a cough peak expiratory flow measurement (CPEF), is strongly associated with extubation success. Furthermore, various cutoff thresholds have been identified and can provide reasonable diagnostic accuracy and predictive power for extubation failure.

These results demonstrate that measurement of the CPEF can be a useful tool to predict extubation failure in patients on MV who have passed a SBT. In addition, the data suggest that this diagnostic modality may reduce ICU length of stay, ICU expenditures, and morbidity and mortality.

## Background

Discontinuation of mechanical ventilation (MV) remains a paramount objective in the daily care of intubated patients in the intensive care unit (ICU). As the disease process responsible for respiratory failure resolves, swift evaluation and action is required on discontinuing MV and removing the endotracheal tube (ETT) as soon as feasible and safe for the patient. Unnecessary delays can lead to numerous complications, such as ventilator associated pneumonia (VAP), ventilator associated lung injury (VILI), atelectasis, pneumothorax, stress gastropathy, arrhythmias, volume retention, and malnutrition [1]. On the other hand, if MV discontinuation is performed prematurely, this may lead to re-intubation which is associated with increased morbidity [2], increased hospital and ICU length of stay, and mortality [3]. Use of protocol bundles [4] and interdisciplinary team [5, 6] approaches to MV liberation have been developed to prevent these attendant complications and also successfully reduce the duration of MV.

Whereas discontinuation of MV refers to the removal of the endotracheal tube, weaning from MV refers to the gradual de-escalation of respiratory support to allow the patient to tolerate an environment without mechanical support. Weaning is achieved when a patient passes a spontaneous breathing trial (SBT). A SBT is accomplished by shifting the patient from full ventilator support to a period of breathing without assistance from the ventilator. A patient passes a SBT by demonstration of appropriate oxygenation and ventilation, hemodynamic stability, and the ability to initiate an inspiratory effort. Guidelines suggest that patients who pass a SBT can be further assessed by parameters such as the mouth occlusion pressure 0.1 s after the onset of inspiratory effort (P0.1/PIMax) [1] and the CROP score (index including compliance, rate, oxygenation, and pressure) [1]. Positive tests for the P0.1/PIMax and the CROP score have been validated to have significant positive likelihood ratios in identifying successful extubation. Further, guidelines recommend routine use of ventilator liberation protocols, early mobilization, and selected use of non-invasive ventilation to prevent extubation failure. However, up to 21% of patients who have passed a SBT may still fail extubation due to excess secretions, inability to clear the airway, impaired neurological status, or laryngospasm [7, 8]..

There is interest in utilizing cough strength, by measuring cough peak expiratory flow (CPEF) during MV weaning, as a metric for predicting successful extubation. The appeal of this measurement procedure is that it is straightforward, inexpensive, portable, easily reproducible, and has the potential to prevent re-intubations.

The morbidity and mortality of patients on MV who fail initial extubation are significant [2, 3], and steps to improve these outcomes are paramount. Furthermore, annual critical care medicine costs have been increasing steadily and have been cited to be as high as 81.7 billion dollars annually [9]. This has relevance in an era of rising health care costs. Therefore, the potential of CPEF in identifying patients at risk of extubation remains a promising area of active research.

## Main text 

### Cough strength

Cough is an innate defensive mechanism that prevents aspiration and clears airway debris. It is a physiological maneuver that requires optimal and coordinated use of the respiratory muscles, airway caliber, and larynx [10]. Kang et al. identified a statistically significant correlation between cough strength, as measured by cough peak expiratory flow (CPEF), and markers of respiratory muscle strength such as the maximal inspiratory pressure (MIP) and maximal expiratory pressure (MEP) [11]. Because the ability to clear the airway of obstructive debris is a requisite for successful liberation from MV, it is reasonable to surmise that CPEF measured prior to extubation may provide useful information regarding extubation failure.

### Peak expiratory flow

**Fig. 1:**
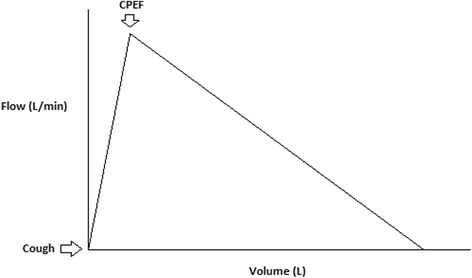
Cough peak expiratory flow. Cough peak expiratory flow (CPEF) measures an individual’s maximum speed of expiration during cough and represents the airflow through bronchi and is inversely proportional to the degree of airway obstruction. It is measured by connecting an electronic peak flow meter to the connector of an endotracheal tube

Peak expiratory flow rate (PEFR) is the maximum flow rate generated during a forceful exhalation, starting from full lung inflation. PEFR reflects large airway flow and depends on the voluntary effort and muscular strength of the patient (Fig. 1). The PEFR has been demonstrated to correlate well with the forced expiratory volume over 1 s (FEV1) [12]. In an ICU setting, a peak flow meter can be improvised to be attached to the opening of an endotracheal tube to measure the CPEF.

### Narrative review

An early use of CPEF was demonstrated by Bach and Saporito in a study of 49 patients with chronic ventilatory failure due to primary neuromuscular disease. A CPEF greater than 160 L/min following decannulation was a discriminating threshold for decannulation failure [13]. Although the results of this study cannot be generalized to a general ICU population, this initial study had stimulated clinical interest in use of this modality.

The first prospective study of a medical ICU population evaluating cough strength during MV weaning was performed by Khaimees et al. Out of 100 study patients, 18 patients had extubation failure within 72 h. A weak cough strength or absent cough, ranked on a semi-objective scale, was associated with a significantly higher likelihood of extubation failure than a patient with a moderate or abundant cough (RR 4.0, 95% CI 1.8–8.9). Moreover, an inability to cough on and moisturize a white card held from 1 to 2 cm from the endotracheal tube (white card test, or WCT) was associated with extubation failure (RR 3.0, 95% CI 1.3–6.7) [14]. Thille et al. also identified that an ineffective cough (graded on a semi-objective scale) is a predictor of extubation failure and was more predictive than delirium or ICU acquired weakness [15]. Duan et al. also demonstrated a strong correlation between CPEF and the semi-objective cough scale (*r* = 0.69, *P* < 0.001) [16]. Hence, the semi-objective cough scale may be useful. However, a major factor limiting generalizability in these studies is the subjectivity of the cough strength evaluation.

Smina et al. designed a similar study instituting an objective measurement of cough strength by measurement of the CPEF. In a prospective study of 95 patients undergoing mechanical ventilation, CPEF prior to extubation was found to be an independent predictor of extubation failure. A CPEF of < 60 L/min was associated with a significantly increased risk of extubation failure (RR 5.1, 95% CI 1.7–15.4, *P* = 0.003) [17]. Further analysis revealed that this CPEF cutoff only reached statistical significance for the primary outcome among a subpopulation of patients with acute physiology and chronic health evaluation II (APACHE II) score of > 24, rapid shallow breathing index (RSBI) < 100, age > 65, or serum hemoglobin of < 10 mg/dL. Importantly, patients that failed extubation had a longer hospital length of stay (median 22 days vs 12 days, *P* = 0.01). Salam et al. performed a follow-up study that affirmed the findings of Smina et al. but cast doubt upon the utility of the WCT. In this study of 88 patients in a medical ICU setting, extubation failure was associated with a CPEF of < 60 L/min (RR = 4.8, 95% CI 1.4–16.2). Contrary to the previous study by Khaimees et al., the presence of a negative WCT was not predictive of extubation failure (RR 2.3, 95% CI 0.8–6.7, *P* = 0.1) [18].

Careful analysis of the data from the previous studies reveals that the mean CPEF rate of patients who failed extubation is around 40 L/min. Analysis of diagnostic accuracies in each of the preceding studies would yield best predictive value by using the 60 L/min cutoff. To better refine the diagnostic accuracy of CPEF, Beuret and colleagues performed a prospective observational study of 130 study patients with this cutoff in mind. Fourteen patients (10.8%) experienced extubation failure, and a CPEF cutoff of <35 l/min predicted this outcome with a sensitivity of 79%, specificity of 71%, positive likelihood ratio (LR) of 2.72, and a negative LR of 0.29 [19].

CPEF has also been studied in the non-medical ICU population. Among burn patients in the ICU, Smailes et al. demonstrated that a CPEF < 60 L/min was highly predictive of extubation failure (RR of 9.1, 95% CI 4.0–20.6, *P* < 0.0001). However, the authors also showed that a high CPEF does not necessarily correlate with extubation success (ρ = 0.38, 95% CI 0.22–0.52, *P* < 0.0001) [20]. In a study by Kutchak et al. of a population of patients on MV for a primary neurological indication, a CPEF of < 80 L/min was demonstrated to predict extubation failure (RR 3.5, 95% CI 2.0–6.7, *P* < 0.001) [21].

Patients who otherwise have successfully weaned but are uncooperative due to either delirium or other psychiatric conditions preclude accurate measurement of CPEF. In this subset of patients, induction of cough with aerosolized saline and measurement of involuntary CPEF (IV-CPEF) can be performed. In a study of 140 patients on MV who had passed a SBT, an IV-CPEF cutoff of 58.5 L/min demonstrated maximal diagnostic accuracy for prediction of extubation success (positive predictive value of 0.930, negative predictive value 0.500, and 95% CI 0.706–0.898) [22]. However, a subsequent study of 115 patients, use of an IV-CPEF cutoff to predict extubation failure did not reach statistical significance [23].

Most recently, Duan et al. demonstrated with an innovative use for CPEF for patients on MV. In a study of 356 patients in a respiratory ICU, consisting largely of patients with COPD exacerbation, they demonstrated that prophylactic use of non-invasive ventilation (NIV) in patients with a CPEF < 70 L/min led to reduced extubation failures compared to non-use of NIV [24]. We had previously praised this elegant study [25] for advancing the literature of the selection of patients with COPD exacerbation for use of NIV in the postextubation period [26, 27].

Despite the encouraging results in the literature, there are some potential shortcomings that prevent widespread adoption. From a practical perspective, measurement of CPEF requires a spirometer, a microbial filter, and a special connector to the ETT. This equipment may not be universally available in all ICUs. From a methodological perspective, these studies are all single center observational studies in specific patient populations with limited external validity. Furthermore, the rates of extubation failure in these individual studies have been fairly small, which limits the power of these conclusions. Another consideration is that several of these studies have taken place in an earlier era where current multidisciplinary approaches to ensuring successful MV liberation and prevention of complications were not as prevalent as they are today.

## Conclusions

CPEF has been shown to add an extra layer of assessment of airway protection in the extubated patient. Performing this evaluation appears to provide a safeguard against extubation failure in patients who have passed a SBT and are otherwise ready for extubation (Table 1). Importantly, these studies also suggest the prospects of decreased ICU length of stay and morbidity and mortality. More methodologically rigorous studies are needed in order to definitively identify the true value of CPEF in this setting. Nonetheless, the current literature does provide optimism that measurement of CPEF can one day be universally adopted and provide cost-effective care in the ICU.

**Table 1:** Summary of included studies and extubation success based on cough peak expiratory flow thresholds

Authors/year	Extubation results	CPEF, mean (L/min)	CPEF threshold established (L/min)	Predictive power
Bach et al. 1996 [13]	43/58 success 15/58 failure	278.0 101.0	160.0	N/A
Smina et al. 2003 [17]	102/115 success 13/115 failure	81.9 64.2	60.0	Extubation failure RR 5.1 (95% CI 1.7–15.4)
Salam et al. 2004 [18]	74/88 success 14/88 failure	79.7 58.1	60.0	Extubation failure RR 4.8 (95% CI 1.4–16.2)
Beuret et al. 2009 [19]	116/130 success 14/130 failure	63.6 36.3	35.0	Extubation failure RR 6.9 (95% CI 2–24)
Su et al. 2010 [22]	118/150 success 32/150 failure	74.0 42.0	58.5	Extubation success RR 0.95 (95% CI 0.93–0.98)
Smailes et al. 2013 [20]	10/125 success 17/125 failure	125.8 74.2	60.0	Extubation failure RR 9.1 (95% CI 4–20.6)
Duan et al. 2014 [23]	95/115 success 20/115 failure	81.3 51.3	62.4	Predicting re-intubation Sensitivity 85.0% Specificity 64.2%
Kutchak et al. 2015 [21]	90/135 success 45/135 failure	115.3 75.8	80.0	Extubation success RR 0.64 (95% CI 0.51–0.83)
Duan et al. 2015 [16]	158/186 success 28/186 failure	74.3 51.7	Semiquantitative cough strength score (SCSS) SCSS 5 (113.7 L/min) SCSS 4 (79.0 L/min) SCSS 3 (57.5 L/min) SCSS 2 (44.7 L/min) SCSS 1 (39.5 L/min) SCSS 0 (38.4 L/min)	Extubation failure RR 1.0 (reference) RR 3.2 (95% CI 0.7–15.7) RR 4.0 (95% CI 0.8–19.1) RR 4.7 (95% CI 1.0–22.0) RR 6.1 (95% CI 1.3–29.0) RR 7.2 (95% CI 1.5–33.8)

## Abbreviations

ACCP: American College of Chest Physicians
COPD: Chronic obstructive pulmonary disease
CPEF: Cough peak expiratory flow
ETT: Endotracheal tube
FEV1: Forced expiratory volume in 1 s
ICU: Intensive care unit
IV-CPEF: Involuntary cough peak expiratory flow
LR: Likelihood ratio
MEP: Maximal expiratory pressure
MIP: Maximal inspiratory pressure
MV: Mechanical ventilation
NIV: Noninvasive ventilation
P0.1/PIMax: Mouth occlusion pressure 0.1 s after the onset of inspiratory effort
PEFR: Peak expiratory flow rate
RSBI: Rapid shallow breathing index
SBT: Spontaneous breathing trial
VAP: Ventilatory associated pneumonia
VILI: Ventilator induced lung injury
WCT: White card test
